# Advances in graphene-based 2D materials for tendon, nerve, bone/cartilage regeneration and biomedicine

**DOI:** 10.1016/j.isci.2024.110214

**Published:** 2024-06-08

**Authors:** Yuxin Gao, Xu Wang, Cunyi Fan

**Affiliations:** 1 Department of Orthopedics, Shanghai Sixth People’s Hospital Affiliated to Shanghai Jiao Tong University School of Medicine, Shanghai, China; 2 College of Fisheries and Life Science, Shanghai Ocean University, Shanghai, China; 3 National Center for Orthopaedics, Shanghai, China; 4 Shanghai Engineering Research Center for Orthopaedic Material Innovation and Tissue Regeneration, Shanghai, China

**Keywords:** Applied sciences, Materials application, Biomedical materials

## Abstract

Two-dimensional (2D) materials, especially graphene-based materials, have important implications for tissue regeneration and biomedicine due to their large surface area, transport properties, ease of functionalization, biocompatibility, and adsorption capacity. Despite remarkable progress in the field of tissue regeneration and biomedicine, there are still problems such as unclear long-term stability, lack of *in vivo* experimental data, and detection accuracy. This paper reviews recent applications of graphene-based materials in tissue regeneration and biomedicine and discusses current issues and prospects for the development of graphene-based materials with respect to promoting the regeneration of tendons, neuronal cells, bone, chondrocytes, blood vessels, and skin, as well as applications in sensing, detection, anti-microbial activity, and targeted drug delivery.

## Graphical abstract

**Figure:**
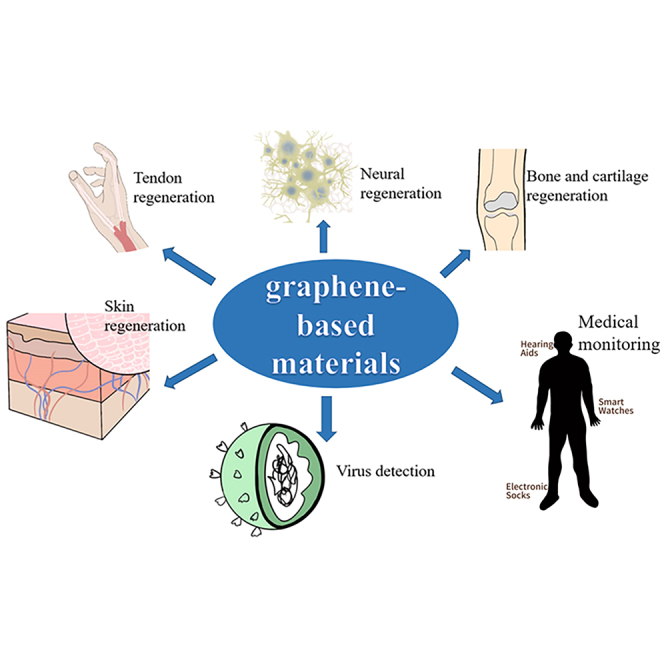

## Introduction

**Figure 1:**
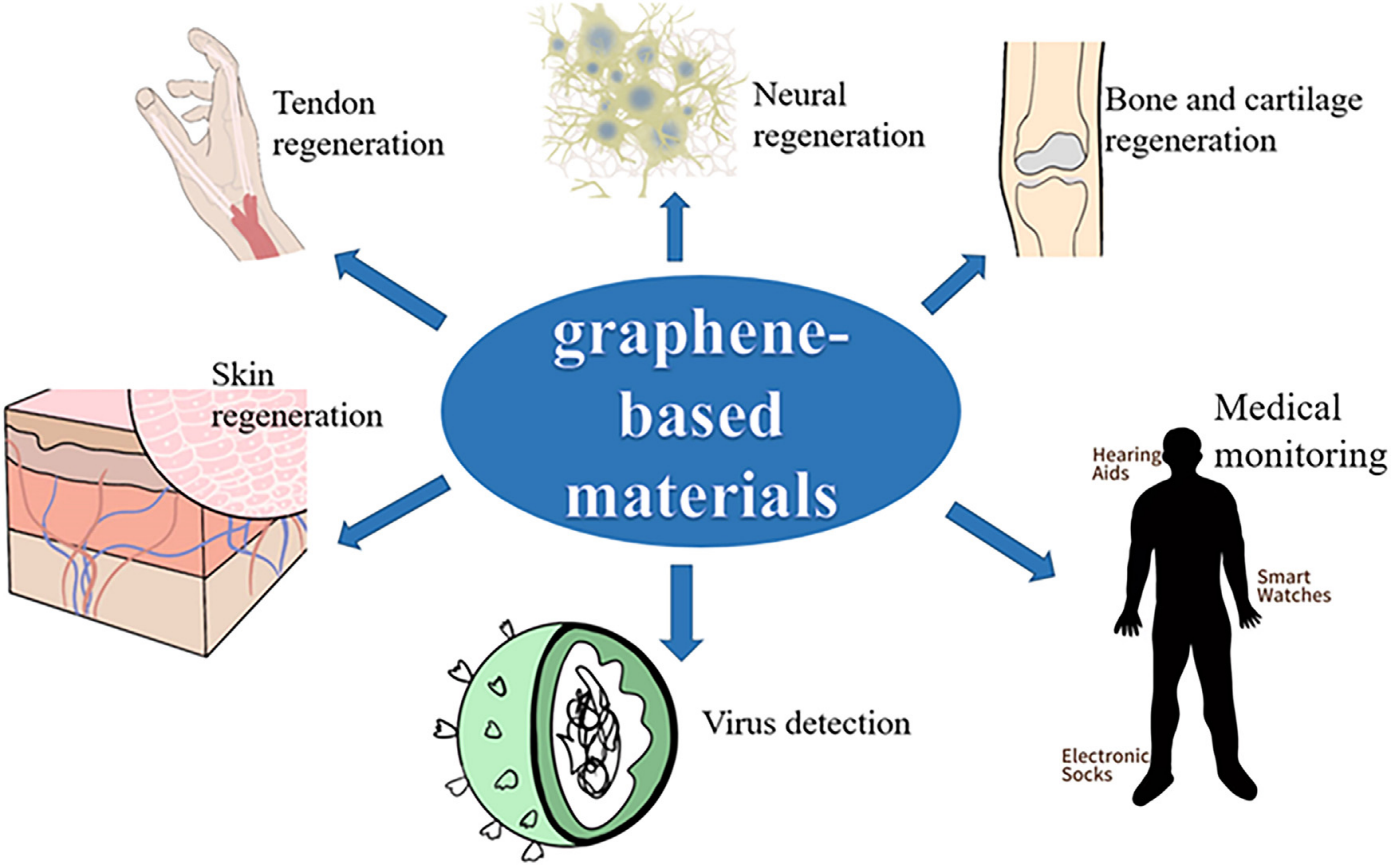
The wide application of graphene-based materials

Since the early 21st century, graphene materials have been successfully obtained, and the preparation of its derivatives has followed.^1^ Due to the layered structure and rich surface chemical properties of graphene-based materials, they have caused extensive research and application in the biomedical field (Figure 1).

Graphene material has a variety of derivatives, such as graphene oxide (GO), reduced graphene oxide (rGO), porous graphene (PG), and so on.^2^^,^^3^^,^^4^ Its structure has the characteristics of large surface area, transport characteristics, easy functionalization, biocompatibility, and adsorption capacity, and so on, and has a wide application prospect in the biomedical field.^5^^,^^6^^,^^7^ Compared with other two-dimensional (2D) materials, graphene materials have the characteristics of high strength, high electrical conductivity, high thermal conductivity, and high light transmittance.^8^ At present, it is used in tissue regeneration and biomedical fields: tendon regeneration, nerve regeneration, skin regeneration, biosensing and medical detection, medical diagnosis, and disease indicators.^9^^,^^10^^,^^11^^,^^12^ To explore the potential of graphene-based materials for biomedical applications, researchers need to explore different approaches to their synthesis, characterization, and application. Although graphene-based materials have many advantages, they still need to be adjusted in specific use scenarios using technical means such as 2D materials engineering to further improve their applicability.

In this article, we discuss the synthesis of graphene-based materials, their applications in tissue regeneration and biomedicine, and their mechanisms of action. The core objective is to review the application cases and principles of action of graphene family materials in the preparation, restoration, maintenance, and improvement of functional constructs of tissue function, as well as in biomedical medical diagnosis, treatment, rehabilitation, care, and so on. The research and development process of different graphene-based 2D materials suitable for various application scenarios and engineering requirements is introduced. The challenges and future work of graphene-based applications in biomedical fields such as tissue regeneration are discussed.

## Graphene family and 2D materials

### Graphene

**Figure 2:**
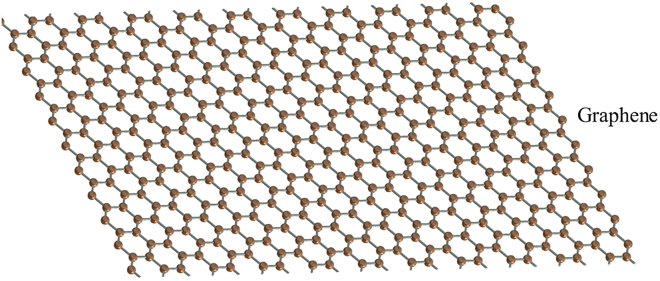
The structure of graphene

Graphene is one of the most important nanomaterials discovered in 2004.^13^ As shown in Figure 2, it is a hexagonal honeycomb structure material arranged by a layer of bonded C atoms with a thickness of 0.34nm.^13^ In it, each carbon atom has three bonds and a planar foreign bond that can bind to adjacent C atoms. This makes the graphene material the thinnest carbide ever made. In addition, graphene has remarkable properties, such as high electrical conductivity, high thermal conductivity, high mechanical strength, excellent optical transmittance and optical absorption.^8^ However, because graphene is made up of only a single carbon atom, there may be some limitations when using graphene.^14^

There are two common graphene preparation methods, namely top-down exfoliation and bottom-up growth.^8^ The top-down approach is based on the preparation of nano/micron-sized materials from bulk materials and typically includes both direct stripping and solid phase stripping. The bottom-up approach involves the preparation of nanoscale materials by reacting atoms or molecules, including wet synthesis and chemical vapor deposition (CVD). Growing on large substrates by methods such as chemical vapor deposition (CVD) is expected to enable large-scale production of graphene.^15^

### Graphene-based materials

Members of the graphene family of 2D materials also include fluorographene, GO, reduced graphene oxide (rGO), PG, graphene nanosheets (GNPs), and graphene nanoribbons.^16^ In addition, there are quantum dot forms, nanowires, and tubes, as well as graphene foam and aerogel forms.^16^ Compared with the original graphene (G), graphene derivatives are more widely used due to chemical modification and have been widely used in tissue regeneration and biomedical engineering.^17^^,^^18^^,^^19^ Among many graphene derivatives, GO and rGO have attracted much attention because of their excellent physicochemical properties.

GO is a material formed by the oxidation treatment of graphene.^8^ During the oxidation process, carbon atoms on the surface of graphene combine with oxygen atoms to form functional groups such as hydroxyl, carbonyl, and epoxy groups, which changes the structure of graphene and makes it easier to handle.^8^ GO has more functional groups than graphene, so it is chemically richer and can react with different compounds.^20^ Due to the presence of functional groups, the surface of GO carries many polar functional groups, so it has good hydrophilicity, can be compatible with polar solvents such as water, and is easy to disperse and suspend. Because GO has good biocompatibility and charge characteristics, it can be used to prepare biosensors, drug delivery platforms, and tissue engineering materials.^21^^,^^22^ In addition, GO has excellent photothermal conversion performance, which can be used for photothermal therapy and photodynamic therapy.^23^

rGO is a material that is reduced from GO and has some of the properties of graphene, such as high electrical conductivity and high mechanical strength.^24^ Compared to GO, the functional groups of rGO are reduced, so it has less oxygen content and higher electrical conductivity,^23^ For example, it can be used to prepare flexible electronic devices, sensors, supercapacitors, and lithium-ion batteries.^25^ Due to its high conductivity and high mechanical strength, rGO can be used to prepare high-performance conductive, and strong materials.^26^ In addition, rGO also has good biocompatibility and biodegradability, and it also has potential application prospects in biomedical and biosensor fields.^27^

GO and rGO are easier to prepare and less costly than graphene. It has good biocompatibility and biodegradability. However, their electrical conductivity and mechanical strength are slightly inferior to that of graphene.^28^

### OTRAS 2D materials

In addition to the graphene family, 2D materials include black phosphorus, Laponite, layered double hydroxides (LDH), transition metal disulfide (TMDs), transition metal oxides, etc.^8^ Black phosphorus, which belongs to the phosphorus group, is a 2D material with a layered structure. Compared to graphene and other biological materials, it will degrade under certain conditions and produce rapid fluorescence.^29^ Therefore, it is very suitable for comparison and fluorescence detectors.^30^ However, compared with graphene, black phosphorus has poor chemical stability and poor mechanical properties,^31^ and it is easy to produce crystal growth in different directions during the preparation of black phosphorus, resulting in differences in its properties and properties in different directions, reducing the reliability and stability of its application.^32^ The chemical structure of transition metal disulfide consists of transition metal and sulfur atoms.^33^ TMDs have a modulated band gap,^33^ excellent optical properties and catalytic activity,^34^ This makes it have a wide application prospect in the field of optoelectronics and catalysis.^35^

## Applications of tissue regeneration

Tissue regeneration explores how damaged or missing tissue can grow again and regain function. Traditional tissue regeneration materials such as gelatin,^36^ polylactic acid,^37^ etc. have the problem of poor mechanical strength, titanium alloy, stainless steel, and other metal and ceramic materials have the problem of poor biocompatibility.^38^ 2D materials such as the graphene family have been widely used for tissue regeneration due to their excellent mechanical properties, large surface area, good biocompatibility, optical transparency, biofunctionability, and controllable electronic and electrochemical properties.

### Tendon regeneration

Tendon is a common injury site of the hand, and it belongs to soft tissue. After a fracture, the regeneration ability is weak, and it is difficult to repair and heal itself.^39^ Therefore, surgical intervention is required. The autograft or allograft used in the past cannot achieve the purpose of clinical treatment due to fatigue, excessive relaxation, or wear.^40^ Although degradable polymers often used in clinical treatment can make up for the shortcomings of the former, their poor mechanical properties make them unable to be used for a long time.^41^ To solve the problems of the above materials, the research has now turned to polymer matrix composites.^42^^,^^43^^,^^44^ This kind of material has higher mechanical properties, thermal properties, electrical properties, and other characteristics than a single polymer material, but also can meet the needs of different applications. The graphene family can improve the physical properties of some materials and promote tissue repair (Table 1).^45^

**Table 1:** Application of graphene-based materials in tendon repair

Fabricating approach	Primary materials	Outcomes	Reference
Chemical cross-linking	PG, PMoS2	PG/PMoS2 hydrogels showed the best healing efficiency, reducing inflammation, accelerating the recovery of Achilles tendon function, and reducing peripheral adhesion and cross-sectional area.	Correia Pinto et al.^47^
Fabrication of nanocomposite thin films	PLA, GNP, HFF-1, CNT-COOH	The addition of PLA enhances the compatibility of GNP and CNT-COOH nanofillers, demonstrating the potential of these carbon-based nanofillers in the fabrication of reinforced synthetic polymer structures for tendon and ligament strengthening.	Saveh Shemshaki et al.^48^
Fabrication of nanofiber matrix	GnPs, PLA	Graphene-based matrices significantly increased myoblastic differentiation and myotube formation in *in vitro*, while inhibiting adipogenesis from adipogenic stem cells. *In vivo* experiments, graphene-based matrices can effectively reverse changes in muscle atrophy, fat accumulation and fibrosis, while improving tendon morphology and mechanical properties.	Font Tellado et al.^49^
Fabrication of nanofiber scaffolds	GO, HA	Graphene-based nanofiber scaffolds show good biomechanical properties, and can also promote the adhesion, proliferation and differentiation of tendon cells, up-regulate the expression of tendon-related genes and proteins, enhance the vascularization and neuralization of tendons, and inhibit inflammatory response and the occurrence of synovitis.	Huangfu et al.^51^
Fabrication of nanofiber membrane	GO, PLGA	GO-PLGA nanofiber membrane can promote tendon and bone integration, increase the formation of new bone and cartilage, improve collagen arrangement and biomechanical properties in rabbit rotator cuff injury model.	Yao et al.^53^

PG: Gallium nitride, PMoS2: Molybdenum disulfide, PLA: Polylactic acid, GNP: HFF-1: Human skin fibroblasts, CNT-COOH: Human skin fibroblasts, GnPs: Graphene nanosheets, HA: Hyaluronic acid, PLGA: Polylactic-glycolic acid copolymer.

It was found that the scaffolds of polyglycerol functionalized RGO composites composed of hydrogels showed good tendon regeneration and anti-inflammatory effects.^46^ In addition, Barzegar et al. also found that this scaffold has the characteristics of accelerating tendon regeneration, good biocompatibility, and simple structure.^46^ Correia Pinto et al. found that graphene nanosheets enhanced c polylactic-based biodegradable membranes without toxicity when implanted in mice.^47^ Shemshaki et al., in a study of large rotator cuff tears in the shoulder joint, found that the integration of graphene nanosheets into aligned poly-1-lactic acid not only reversed muscle degeneration *in vivo* but also inhibited fat formation in fat-derived stem cells to reduce fat accumulation and muscle atrophy.^48^ In addition, tendon repair sometimes requires the establishment of tendon-to-bone reconnection. The attachment point is a key part of tendon repair.^49^ Previous studies have also shown that mesenchymal stem cells proliferate rapidly and can differentiate into osteoblasts when induced by GO.^50^ Moreover, the development process of the tendon-bone attachment site is similar to that of intrachondral bone formation. Therefore, the osteogenic ability of biomaterial scaffolds is crucial for enhancing tendon/ligament-bone integration.^51^ Su et al. promoted the binding of tendons to the bone by doping Goene with a polylactic-glycolic acid nanofiber membrane.^52^

### Neural regeneration

Nerve regeneration is a technique that uses a variety of advanced technologies to restore the sensory and motor functions of patients with nerve injury as much as possible.^53^ Traditional materials used for nerve regeneration include myelin sheath, human extracellular matrix, synthetic porous tube, collagen, and other natural biological materials, as well as metal wires.^54^ But they all have poor biocompatibility and are easy to cause immune rejection. Lack of sufficient growth factors and nutrients to support nerve regeneration; Poor strength, unable to provide effective mechanical support; Problems of difficult preparation and slow biodegradation.^55^^,^^56^^,^^57^

However, some graphene-based materials can better solve these problems. Previous studies have shown that graphene, as an excellent cell adhesion layer, can not only promote blood vessel differentiation of human neural stem cells after nerve injury but also promote neuronal differentiation, axon regeneration and re-myelination.^58^^,^^59^^,^^60^^,^^61^ In the treatment of peripheral nerve injury, artificial nerve guided catheter (NGC) has been widely studied for structural and functional restoration of large nerve defects. Wang et al. made conductive hydrogel-based NGC by polymerizing GO and gelatin methacryloyl (GelMA) and subsequently chemically reducing it to eventually form r (GO/GelMA).^62^ In addition, the study found that the Go-modified composite scaffold has a similar healing ability to autologous grafts *in vivo*, and the biomimetic multi-channel sponge-containing artificial nerve guide catheter (MCS-NGC) made of GO’s blend nanofibers can promote sciatic nerve regeneration *in vivo*.^63^ Zhang et al. demonstrated that GO significantly accelerates the collective migration of Schwann cells (SCs) and the migration of SCs from their spheres.^64^ Conductive materials can promote neurite and axon growth through their electrical stimulation.^65^ rGO has good electrical conductivity (8.7 ± 1.6 mS cm-1). rGO also has a low Young’s modulus (57 ± 13 kPa), low impedance over a wide frequency range, flexibility, durability (up to 500 compression cycles), and permeability.^66^ Melatonin can reduce inflammation and oxidative stress in regenerating nerves.^67^ Jiang et al. using the combination of rGO and melatonin can not only reduce the oxidative stress caused by injury but also improve the attachment and proliferation of nerve cells.^68^

Other materials, such as nanodiamonds, hydrogels, and polycaprolactone also have a repairing effect on nerve damage. Composites made from their combination with graphene-based materials may have better results in nerve repair.^69^^,^^70^^,^^71^

GO also plays a huge role in the treatment of neurodegenerative diseases, especially Alzheimer’s disease. GO’s inherent fluorescence quenching ability can be applied to the detection of Alzheimer’s disease.^72^ Graphene compounds can protect nerves and enhance nerve production in the body and are potential drugs to treat Alzheimer’s disease.^73^ It is worth noting that the MoS2 complex has also been found in studies to have a therapeutic effect on Alzheimer’s disease.^74^

### Skin regeneration

Skin is one of the largest organs of the human body, and skin tissue regeneration refers to the process of re-growth and repair of damaged skin tissue by the body’s repair ability after skin injury.^75^ Skin tissue regeneration is usually divided into three stages: inflammation, hyperplasia, and reconstruction.^75^ In skin tissue repair, angiogenesis is a key step in the wound healing process, providing adequate oxygen and nutrients to the wound area.^76^ However, traditional clinical interventions are insufficient to stabilize the formation of the vascular system to support wound healing. Graphene-based nanomaterial (GBN) wound dressings in thin film or hydrogel form have shown great application prospects.^77^

GBN itself can promote wound healing, while providing bactericidal ability, destroying the DNA structure of the microbial membrane and preventing proliferation, thereby improving the wound healing rate.^77^ Studies have shown that low concentrations of GO and rGO stimulate the synthesis of intracellular reactive oxygen species and active nitrogen, thereby inducing angiogenesis. And fibrous membranes containing 1.5% and 2% GO maximize wound healing.^78^ rGO-loaded nanocomposite scaffolds have greater fluid absorption capacity and can promote angiogenesis, collagen synthesis, and deposition in therapeutic wounds.^79^ GO composite membrane has better mechanical properties and stronger water retention, which can regenerate skin without scars.^79^ In addition, the introduction of GO can give hydrogels high ductility and adhesion and thus can promote angiogenesis and wound healing.^80^

While the GO/rGO complex enables efficient healing of skin wounds, it can also effectively enhance the antibacterial ability of wound dressings due to its unique photothermal effect, thus significantly reducing the risk of wound infection.^75^^,^^81^^,^^82^^,^^83^^,^^84^ Therefore, it is necessary to study graphene-based composites in the repair of skin tissue.

### Bone and cartilage regeneration

Bone and cartilage regeneration is a complex multi-cellular and multi-factor targeted biological process, which requires close collaboration between mesenchymal stem cells, bone/cartilage forming cells, and bone/cartilage absorbent cells to achieve tissue regeneration.^85^ Factors such as regulation of cell activity, provision of growth scaffolds, optimization of mechanical properties, and regulation of the microenvironment are all crucial for promoting the regeneration of bone and cartilage tissue.^85^

Osteoblasts play a crucial role in bone tissue regeneration, promoting the recovery and health of bone tissue through bone formation, bone reconstruction,^86^ and bone metabolism (Table 2). Studies have shown that GO can up-regulate related markers, so GO may promote the activity of bone tissue.^87^ Moreover, it can enhance the attachment and proliferation of human fetal osteoblast (hFOB) cells, that is, GO accelerates osteogenic differentiation and new bone formation.^88^ In addition, GO composites can also promote osteogenic differentiation of human adipose-derived stem cells (hASC), and improve hASC attachment and cytoskeleton formation. The mechanical properties of the material were significantly enhanced to prove the feasibility of GO composites in bone tissue engineering applications.^89^^,^^90^^,^^91^ In human articular cartilage repair, the GO content of GO nanocomposites was upregulated (increased by 0.2%GO), and the proliferation of chondrocytes was enhanced.^92^ Therefore, GO is currently widely used in bone/cartilage regeneration scaffolds, such as Go-alginate composite scaffolds,^93^ Go-polycaprolactone composite scaffolds,^94^^,^^95^ Go-chitosan scaffolds,^92^ Go-gelatin scaffolds^96^^,^^97^ and RGO-gels.^98^

**Table 2:** Application of GO derivatives in bone tissue repair

Fabricating approach	Materials	Outcomes	Reference
Modified Hummer, Ultrasonication	UGO suspension	UGO can induce bone regeneration and skin tissue regeneration to promote bone and skin wound healing.	Thangavel et al.^79^
Modified Hummer, Ultrasonication	GO, NIPAAm, IA, mPEG, MA	Go-based hydrogels promote the proliferation of hDPSCs and differentiation into osteoblasts	Khan et al.^80^
Ultrasonication	DMF, CNT, GO	GO composites enhanced hASC adhesion and cytoskeleton formation	Esmaeili et al.^81^
Electrospun	PCL, GO, DMF, MIT, DCM	GO significantly enhances the mechanical properties of the material to demonstrate the feasibility of GO composites in bone tissue engineering applications	Jian et al.^82^

NIPAAm: N-Isopropylacrylamide, IA: Itaconic acid, mPEG: Polyethylene glycol methyl ether, MA: Maleic anhydride, DMF: Dimethylformamide, CNT: Carbon nanotube, PCL: Poly(ε-caprolactone), MIT: Methylthiazolyldiphenyl-tetrazolium bromide, DCM: Dichloromethane.

In the process of bone remodeling, in addition to osteoblasts and osteoclasts, bone mineralization is caused by external calcium,^99^ and phosphorus metabolism also plays an important role.^100^ 85% of phosphorus is present in bones and teeth in the form of hydroxyapatite,^101^^,^^102^ which is necessary for maintaining bone mechanical strength and inducing bone regeneration.^103^^,^^104^^,^^105^ Black phosphorus (BP) has good degradability,^106^ photo-controlled release,^107^ and photothermal conversion ability in bone tissue regeneration.^107^ These excellent properties make BP as promising as graphene-based materials in bone tissue repair.

Synthetic glucocorticoids (GCs) can treat chronic inflammatory and autoimmune diseases in children, but the mechanism by which they inhibit angiogenesis by inhibiting the formation of osteoclasts at the stem end has an adverse effect on growing bone.^108^ Graphene-based materials can promote angiogenesis.^59^ It may be possible to combine the osteogenic activity of H-type vessels to counteract the adverse effects of GCs.^109^ In addition to tendon regeneration, nerve regeneration, skin regeneration, and bone/cartilage regeneration, the graphene family and other 2D materials can also be used for tissue repair such as brain tissue regeneration.^18^^,^^110^ In the process of using these 2D materials, it has been found that they can activate some signaling pathways. For example, Liu et al. found in their study on the intestinal toxicity of GO in mice that GO can induce ROS-dependent apoptosis of human intestinal epithelial cells *in vitro* by activating the AMPK/p53 signaling pathway.^111^

## Applications of biomedical applications

Biomedicine plays an important role in medical diagnosis, treatment, rehabilitation, nursing, and other aspects, making medicine more accurate and intelligent, and greatly improving the level of human health.^112^ It has also promoted the innovation and development of new materials and new technologies. Biomedical applications place very high demands on materials, as these materials will come into direct or indirect contact with living organisms and may have an impact on biological tissue and life health.^113^^,^^114^ Therefore, biomedical materials need to have good biocompatibility, biodegradability, and bioactivity. Graphene-based materials provide a direction for biomedical research.

### Biosensing and medical monitoring

Biosensing and medical monitoring are mainly the use of various sensing technologies to detect and monitor specific components or physiological parameters in biological samples to achieve rapid, highly sensitive biological analysis and medical diagnosis.^115^ Compared with traditional biochemical analysis, biosensing, and medical monitoring have the advantages of high sensitivity, real-time response, ease of use, and low cost.^116^ It is widely used in disease prevention, clinical diagnosis, drug research and development, food safety, environmental monitoring, and other fields.^117^^,^^118^ Graphene family materials (G/rGO/GO) can respond to physical and chemical stimuli, convert them into electrical signals, can achieve a variety of sensing functions, so can be used to create wearable sensors, and for biophysical signals, such as the detection of human movement, pressure, touch, vibration, temperature, electrooculogram, electroencephalogram, electrocardiogram and electromyography.^119^

The graphene film prepared by the CVD method has an electrical conductivity of up to 9000 cm2·V^−1^·s^−1^^120^ and the resistivity of the graphene film is only 31Ω/◻.^121^ rGO also has excellent high conductivity.^122^ And the graphene material can be integrated with different flexible substrates to make flexible health monitoring sensors.^123^

GO can be chemically modified and its affinity with water molecules can be used to make humidity sensors.^124^ Previous studies have shown that water embedding in 2D material layers can significantly improve the response time and sensitivity of GO. For example, at 40% relative humidity (RH), the response time of GO nanocomposite is 82.67 times that of normal conditions, and its sensitivity is 95.7 times that of normal conditions at 60%RH.^125^ Wang et al. found that the GO humidity sensor has a frequency change of 103 kHz/%RH at low humidity (30–60%RH).^124^ In addition, Wu et al. demonstrated that a GO-based humidity sensor has ultra-high sensitivity at room temperature, with a response time of 3 s faster than normal conditions and a wide detection range of 8–95% RH. Singh et al. used GO for plasmon-resonance-based biosensors to further improve their sensitivity.^126^ However, at high temperatures, the sensitivity of the GO humidity sensor decreases, indicating that the influence of temperature on the sensor cannot be ignored when applied.^127^

Due to the low technical threshold and large market demand, body motion sensors based on graphene materials are widely studied and have been applied commercially. The use of screen printing methods can be large-scale, low-cost deposition of graphene on textiles to achieve the manufacture of wearable electronic devices, but the performance and versatility of this device are limited. In short, graphene-based materials offer broad application prospects for wearable sensors.

### Medical diagnosis and disease treatment

#### Virus detection

So far, researchers have developed a variety of virus detection technologies, which can generally be divided into three categories, the detection of viral particles (virus particles and viral proteins), the detection of antibodies, and the detection of viral nucleic acids.^128^ However, the current virus detection technology has some limitations such as low specificity, lack of sensitivity, and complex operation. Therefore, there is an urgent need for simple, specific, sensitive, and inexpensive virus detection reagents to improve detection efficiency. Graphene-based materials have attracted the attention of researchers. Roberts et al. developed a graphene-composite biosensor for the detection of the Japanese meningitis virus and avian influenza virus,^129^ and Chan et al. reported a microfluidic integrated rGO FET for the detection of the H5 N1 influenza virus.^130^ In addition, graphene-based biosensors were used to detect the Zika virus,^20^ graphene field effect tubes were used to detect dengue virus RNA,^131^ and molecular imprinting was performed on MoS2 monolayers to achieve specific identification of viral proteins.^132^ Reddy et al. used NiO-rGO/MXene nanocomposites to make a nano biosensor to detect active influenza viruses and viral proteins.^133^ However, at present, graphene-based virus detection technology is rarely used in clinical practice, and some problems in practical applications may not be considered.^134^

#### Bioimaging

Biomedical imaging is a technology that uses various physical principles and technical means to obtain the morphological and functional information of molecules, cells, tissues, and organs in living organisms. Its key advantages are visualization, non-invasiveness and high sensitivity, which can be used for early diagnosis of diseases. Therefore, biomedical imaging technology has the characteristics of good biocompatibility, high imaging contrast, strong positioning ability, and high quantum efficiency for imaging materials.^135^

2D materials are widely used in biomedical imaging because of their unique physical and chemical properties. Graphene quantum dots (GQD) and some TMD materials (such as MoS2, WS2, etc.) have strong fluorescence emissions and can be used as excellent optical contrast agents.^136^^,^^137^ GQD consists of a single layer to several layers of graphene sheets with a transverse size <10 nm.^138^ At present, GQD has been studied and applied to fluorescence imaging, two-photon imaging, magnetic resonance imaging, and dual-mode imaging.^135^ Due to its small size, GQD effectively penetrates the cell’s nucleus. Hong et al. detected HeLa cell nuclei by tracking the blue fluorescence of GQD during fluorescence imaging,^139^ Based on the water solubility, excellent biocompatibility, and non-toxicity of GQD, Ding et al. A therapeutic diagnostic nanoparticle based on GQD loaded with doxorubicin (DOX) was developed,^140^ Kuo et al. developed amino-functionalized N-GQD (amino-*n*-GQD) as a dual-mode reagent for antimicrobial PDT.^141^ In addition, GQD-based dual-mode imaging can introduce multiple imaging functions into a single reagent, which can avoid its stress on the body and can enable the contrast agent to be effectively removed.^135^

### Antibacterial, antiviral, and antifungal action

It is important to understand the interactions between graphene-based materials and microorganisms such as bacteria, viruses, and fungi. However, due to the variable intrinsic properties of graphene materials, its potential mechanism of action as an antiviral and antifungal drug remains unclear. This section describes various hypothesized mechanisms including nanoknives, wrapping, oxidative stress, and mold stress.

The nanoknife mechanism, also known as the “insertion mode”, is a mode that defines how easy it is for microbes to enter the membrane and kill cells because the edges of the graphene-based material are as sharp as an atomic knife.^142^^,^^143^ According to relevant studies, graphene-based materials, due to their sharp edge properties, allow DNA or RNA to flow in the cytoplasm, resulting in cell death.^144^^,^^145^ In this mechanism, the graphene material layer thickness and hydrophilicity may affect the degradation of membrane integrity by single-celled microorganisms.^146^ In addition, the orientation Angle between the graphene-based material and the microorganism also affects the antibacterial efficiency. Under this hypothesized mechanism, if the GMs edge is parallel to the surface of the microbial membrane, it may not work. However, in the parallel mode, the voids on the graphene surface may destroy the phospholipid structure of the microorganism and lead to the death of the cell.^147^^,^^148^

According to simulation studies of membrane interactions and lipid extraction, graphene and GO may penetrate the surface of microorganisms to achieve the effect of sterilization, i.e., membrane stress.^148^^,^^149^ According to the results of Tu et al., the van der bond and the hydrophobic interaction may enhance the antibacterial effect.^150^ In addition to the cell membrane, damage to phospholipids also mimics cell death. This may be because of the hydrophobic contact between lipid molecules and GO. This may be because of the hydrophobic contact between lipid molecules and GO. Kong et al. used molecular dynamics simulations to investigate the transport mechanisms of GQD across cell membranes to interact with cell surfaces and lipids.^151^ According to this simulation, the cells can automatically penetrate locally at the corners and prominences of the film, and then spontaneously diffuse along the edges of the graphene to complete the penetration. The other major mechanism of graphene is defined as the “oxidative stress” mode. Oxidative stress due to the formation of reactive oxygen species (ROS) within the cell leads to degeneration of the cell membrane and triggers cell necrosis.^142^^,^^152^ It is well known that when the production and removal of reactive oxygen species are out of balance, cells are unable to take advantage of their built-in repair systems to withstand the accumulated oxidative damage. Furthermore, certain metal oxides contained in nanocomposites, such as CuO2, help to promote ROS formation in graphene composites with the help of light.^149^^,^^153^ These photocatalytic materials increase the biocidal effect of the polymer graphene hybrid. Graphene nanocomposites are composed of some polymers, such as quaternized chitosan,^154^ which can increase the production of reactive oxygen species when an electric potential is applied, thus inhibiting microorganisms. Graphene-silver nanocomposites improved the antibacterial effect through synergistic effect, especially for enterobacter and Staphylococcus aureus showed excellent antibacterial activity.^143^^,^^144^^,^^145^

### Delivery of chemotherapeutics and biological drugs

Graphene and its derivatives are among the first 2D materials to serve as drug delivery vehicles due to the ability of delocalized pi electrons on their surface to anchor cancer drugs using PI-PI interactions.^155^ In addition, it allows surface functionalization of graphene surfaces, particularly in GO and reduced GO, to load drugs via covalent bonds. GO has hydroxyl and epoxide functional groups, which can perform effective physical adsorption or chemical adsorption for drugs with high biocompatibility and stability, so it has a wider range of applications in drug delivery.^156^

In addition, graphene and its derivatives can also be used for biological drug delivery. It can be used in the treatment of genetic diseases such as viral, cardiovascular, cancer, Parkinson’s disease, etc.^20^^,^^157^ Graphene can protect genes from lysosomal degradation and deliver them to target sites while being non-toxic and non-immunogenic. The ionization calculation is very fragile: because the negative charge is limited, it decomposes quickly. GO is negatively charged due to the hydroxyl group on its surface, so it can be functionalized by positively charged polymers such as PEI and PEG.^158^ Positively charged polymers such as PEI-rGo have been functionalized to form complexes of siRNA domains and become excellent platform materials for gene silencing.^159^

## Conclusions

This article focuses on the structure and properties of 2D materials, especially the graphene family, as well as the applications of graphene-based materials in tissue regeneration and biomedicine.

In tendon regeneration, graphene can be used as an enhancer for many polymer-based composites to increase the strength, stiffness, and toughness and enhance the biocompatibility and biofunctionality of the polymers. In nerve regeneration, G/GO/rGO can promote nerve cell regeneration and detect and treat neurodegenerative diseases such as Alzheimer’s disease due to its unique electrical coupling. In skin regeneration, graphene-based composites can not only promote skin angiogenesis and wound healing but also play a certain bactericidal role in preventing wound infection. In bone and cartilage regeneration, GO can promote the differentiation of osteoblasts and the formation of bone, so GO composite materials can be used as scaffolds for the repair and regeneration of bone and cartilage. It is worth noting that black phosphorus has also been studied for bone and cartilage regeneration applications due to its good degradability, photothermal properties, etc. In biosensing and medical monitoring, graphene-family materials can respond to physicochemical stimuli and be converted into electrical signals to achieve various sensing functions. Because of its affinity to water molecules, GO can be used as a humidity sensor to improve its sensitivity and shorten its response time. In medical diagnosis and disease treatment, the specificity and accuracy of virus detection can be improved due to the high specificity and sensitivity of graphene-based materials. GQD are often studied and applied to different biological imaging because of their water solubility, excellent biocompatibility, and non-toxicity. In terms of antibacterial, graphene materials can effectively resist bacteria, fungi, and microorganisms due to the three mechanisms of nanoknives, wrapping, oxidative stress, and mold stress. Finally, graphene materials can deliver drugs because of the ion domain on their surface.

However, these are mostly *in vitro* experiments. Data on the long-term safety, biodegradability, metabolic pathways, and long-term stability of graphene-based materials *in vivo* are lacking in both tissue regeneration and biomedical fields. Therefore, more clinical trials are needed in the future.

In short, graphene-based materials have a huge role in the field of tissue regeneration and biomedicine, but how they can be combined with other materials into composite materials that can be applied to clinical treatment still needs to be further explored.
